# Climate Change, Drought and Human Health in Canada

**DOI:** 10.3390/ijerph120708359

**Published:** 2015-07-17

**Authors:** Anna Yusa, Peter Berry, June J. Cheng, Nicholas Ogden, Barrie Bonsal, Ronald Stewart, Ruth Waldick

**Affiliations:** 1 Environmental Health Program, Health Canada, 180 Queen St. West, Toronto, ON M5V 3L7, Canada; 2 Climate Change and Health Office, Health Canada, 269 Laurier Ave. West, Ottawa, ON K1A 0K9, Canada; E-Mail: peter.berry@hc-sc.gc.ca; 3 Sherbourne Health Centre, 333 Sherbourne St., Toronto, ON M5A 2S5, Canada; E-Mail: june.cheng@medportal.ca; 4 Centre for Food-Borne, Environmental and Zoonotic Infectious Diseases, Public Health Agency of Canada, 3200 Sicotte, P.O. Box 5000, Saint-Hyacinthe, QC J2S 7C6, Canada; E-Mail: nicholas.ogden@hc-sc.gc.ca; 5 Watershed Hydrology and Ecology Research Division, Environment Canada, 11 Innovation Blvd., Saskatoon, Saskatchewan S7N 3H5, Canada; E-Mail: barrie.bonsal@ec.gc.ca; 6 Department of Environment and Geography, University of Manitoba, 70A Dysart Road, Winnipeg, MB R3T 2N2, Canada; E-Mail: ronald.stewart@umanitoba.ca; 7 Environmental Health, Agriculture and Agri-Food Canada, 960 Carling Avenue, Ottawa, ON K1A 0Z2, Canada; E-Mail: ruth.waldick@agr.gc.ca; 8 Department of Geography and Environmental Studies, Carleton University, 1125 Colonel By Drive, Ottawa, ON K1S 5B6, Canada; E-Mail: ruth.waldick@carleton.ca

**Keywords:** climate change, drought, adaptation, Canada, human health impacts

## Abstract

Droughts have been recorded all across Canada and have had significant impacts on individuals and communities. With climate change, projections suggest an increasing risk of drought in Canada, particularly in the south and interior. However, there has been little research on the impacts of drought on human health and the implications of a changing climate. A review of the Canadian, U.S. and international literature relevant to the Canadian context was conducted to better define these impacts and adaptations available to protect health. Drought can impact respiratory health, mental health, illnesses related to exposure to toxins, food/water security, rates of injury and infectious diseases (including food-, water- and vector-borne diseases). A range of direct and indirect adaptation (e.g., agricultural adaptation) options exist to cope with drought. Many have already been employed by public health officials, such as communicable disease monitoring and surveillance and public education and outreach. However, gaps exist in our understanding of the impacts of short-term *vs.* prolonged drought on the health of Canadians, projections of drought and its characteristics at the regional level and the effectiveness of current adaptations. Further research will be critical to inform adaptation planning to reduce future drought-related risks to health.

## 1. Introduction

The Intergovernmental Panel on Climate Change (IPCC) recognizes that climate change and the altered frequencies of associated extreme events such as drought and floods are expected to have negative impacts on human health [1]. For Canada, projections call for more frequent and severe extreme weather events including drought [2]. Research into the health impacts of drought and how climate change may enhance these effects is still in its infancy. A number of key health concerns related to climate change including food-, vector- and water-borne diseases may be exacerbated by drought. In addition, there is the potential for broad and lengthy exposure of Canadians to these episodes. There is particular concern about changes in the frequency, duration and severity of drought in the future due to climate change [3]. For these reasons, understanding of drought-related health risks is a climate change research priority. This paper reviews current understanding of the health impacts associated with drought that can be anticipated with climate change in the Canadian context drawing on Canadian, U.S. and international literature sources. It assesses the state of knowledge about adaptation options to protect health in the face of drought conditions and suggests future research directions to support adaptation efforts by health sector officials.

### 1.1. Canadian Drought Features

There is currently no single, clear, consistent definition of drought. For the purposes of this discussion, drought will be considered a prolonged period of abnormally dry weather that depletes water resources for human and environmental needs [4,5]. Droughts stress water availability by lowering lake and reservoir levels, reducing stream flows, diminishing groundwater supplies and depleting soil moisture. Droughts can also create major environmental hazards such as reduced water quality, wetland loss, soil erosion and degradation, ecological habitat destruction and increased risk of wildfires. Prolonged, large-area droughts are one of Canada’s major natural disasters and can have large scale impacts on various sectors including human health and society, agriculture, forestry, industry, municipalities and recreation [4]. Droughts differ from other disasters (e.g., floods) since they have longer durations, lack easily identified onsets and terminations and their recurrence is practically certain in most environments including many regions of Canada [6]. Climate anomalies, lasting from a month to one or more years, are the root of most droughts, although human impacts on resources and climate and the changing demand for water are major contributing factors [7].

There is no one “straightforward” manifestation of drought. Each drought manifests itself differently depending on factors, such as area affected, duration, intensity and society’s capability to adapt to water shortages. Thus, although the general features of drought tend to be similar, the actual features vary between droughts and evolve within a single drought. This complexity may lead to varying impacts [8,9]. Some precipitation normally occurs even within a period of drought. It is not necessarily the case that the number of days with precipitation actually decreases during a drought; the relative lack of large precipitation events can for example be a contributor to drought formation. When it occurs during drought, precipitation tends to come in small amounts and to be highly scattered spatially due to convective processes, although more widespread rain can fall as well [9]. Sometimes, droughts can even be linked with heavy precipitation events. This can occur when the atmospheric forcing that is causing reduced precipitation in one region results in nearby areas receiving more rain than usual. The boundary between these regions can shift, thereby creating a situation in which conditions in some locations can alternate between both extremes. In some cases, the “end” or significant reduction of a drought can come in the form of a single heavy precipitation event. For example dry conditions in 2002 across the Prairies were alleviated by a torrential multi-day event [10].

Surface air temperatures are often high/elevated during drought, although this is not always the case. For some long duration droughts, there are periods when temperatures are below normal [11]. Droughts can also be windy or calm. Moderate to high winds can lead to erosion and transport of desiccated soil, although the number of these instances has generally declined, in part due to altered farming practices.

### 1.2. Historical Occurrence of Canadian Droughts

Canada is considered a country with abundant water resources; however, these resources and the demands on them are distributed unevenly across the country [12] resulting in varied vulnerability to, and impacts from, drought. For example, 98% of the Canadian population resides in the South, which only provides 38% of the water yield (Water yield is the volume of freshwater resources for a defined geographic area and time period. It is an estimate of the amount of renewable water and non-renewable glacial melt) [12]. In the North, the per capita water yield is 98 times greater than in the South [12]. The water yield in the Prairies is a fraction (3%–12%) of that in the Great Lakes, Maritime Coastal or Pacific Coastal drainage regions and decreased between 1971 and 2004 [12].

During the 20th century, meteorological measurements have shown significant warming during all seasons but the greatest rates have been during winter and spring. They have also revealed general increases in precipitation, although considerable decadal-scale variability is present over many regions of the country. For example, Zhang *et al.* [13] determined that mean annual air temperature has increased by an average of 0.9 °C over southern Canada between 1900 and 1998 with the greatest rates seen in the West during the winter and spring (southern Canada broadly refers to south of 60N; an exception is [314] and mention in [315] where southern Canada refers to south of 55N. See Figure 5 in [4] for additional details. For Statistics Canada discussions, see [12] for a description of standard drainage area classifications). Subsequent analysis extending the target period to 2010 confirmed the warming has continued and even accelerated with an increase in the mean annual temperature of 1.5°C for southern Canada over this longer period [16]. Zhang *et al.* [13] also found that annual total precipitation increased significantly from 1990–1998 over most of southern Canada with the exception of southern Alberta and Saskatchewan. Analysis expanded to the period 1900 to 2009 also found an 8.7% increase in annual rainfall for southern Canada [17]. Snowfall mainly increased in northern Canada while at the same time decreasing significantly in the Southwest for the period 1950–2009 [17].

An analysis of changes in extreme temperature indices over southern Canada from 1900–2003 and the entire country from 1950–2003 found that over most of the country, there was an increase in the number of warm days where the maximum temperature (>90th percentile) and the number of warm summer days (maximum temperature >25°C) during the last half century [18]. The number of hot days (>30°C) and summer warm days (>90th percentile) also increased over all of Canada, but were only significant in southwestern regions [19]. The analysis revealed little change in the number of summer warm spells. An examination of changes in extreme temperature and precipitation indices over southern Canada from 1900–2003 and the entire country from 1950–2003 found a decrease in the consecutive number of days with no measurable precipitation in British Columbia and Atlantic Canada. Trends in drought occurrence and severity in Canada are often monitored using meteorological drought indices, such as the Standardized Precipitation Index (SPI), the Palmer Drought Severity Index (PDSI), and the Standardized Precipitation Evapotranspiration Index (SPEI) [4]. These indices are designed to assess regional precipitation anomalies (the SPI), or a region’s water balance using both precipitation and temperature data (PDSI and SPEI), to account for evapotranspiration effects. Results from trend investigations incorporating these various drought indices have shown variable results over Canada, often being dependent on the time period used in the trend calculation (mainly due to the aforementioned decadal-scale variability in precipitation). However, an increase in drought (i.e., a decreasing Palmer Drought Severity Index (PDSI) trend) over most of the country was observed between 1950 and 2002 [4].

Western Canada, notably the southern Canadian Prairies and interior valleys of British Columbia, are more susceptible to drought because they lie in the lee of major mountain ranges and, as a result, generally receive little precipitation but with high variability [20]. During the past 100+ years, several long-duration droughts have occurred in western Canada. The Canadian Prairies have experienced large-area, multi-year dry episodes during the 1890s, 1930s, late 1950s/early 1960s, 1980s and more recently from 1999–2005 [21,22]. During the notable drought period from 2001–2002, below average stream flows were reported across the country extending from western Canada to Ontario and the Atlantic Provinces [23]. Water levels of most closed-basin lakes have declined throughout the 20th century across southcentral Alberta and Saskatchewan in western Canada, although levels have increased in the past few years [24].

Farther east, droughts do occur in southern Ontario and Quebec, although these events are usually shorter, smaller in area, less frequent and less intense [20]. Nonetheless, there have been major drought occurrences in the East during the last century [25] resulting in severe impacts, including low water levels in the Great Lakes [26]. Between 1920 and 1999, southern Ontario experienced major droughts in 1930, 1933, 1934, 1936, 1963, 1998, and 1999 [25]. Ontario was also impacted by drought in 2001–2002 [23]. Great Lakes’ water levels have shown considerable decadal-scale variability during the 20th century with no evidence of any long-term trend. However, lower Great Lakes’ water levels have coincided with the droughts of the 1930s, early 1960s and from early 2000 to the present [20].In 2011, water levels in the Great Lakes–St. Lawrence Rivers system reached record low levels and some activities/sectors in Ontario watersheds were asked to reduce water consumption under the Ontario Low Water Response Plan (OLWR)[23]. Déry *et al.* [27] found that freshwater discharge rates decreased over a period of 37 years for the majority of key rivers feeding the Hudson, James and Ungava Bays. This watershed is fed by water from five provinces, the territory of Nunavut andfour U.S. states, covering an area that is equivalent to one-third of Canada [27]. Along the East Coast, droughts in the Atlantic provinces are infrequent. However, a side effect of their rarity is that these regions have lower adaptive capacity to deal with droughts, making the region more susceptible to drought impacts [28]. The Atlantic provinces were affected by the drought of 2001–2002, with river flows in the Atlantic Provinces dropping to a 20-year low [23]. An analysis of meteorological indices (southern Canada 1900–2003; entire country 1950–2003) indicated a decrease in the number of days with no measurable precipitation in Atlantic Canada [18]. The context surrounding this shift will be discussed further below.

### 1.3. Future Droughts

All Global Climate Models (GCMs) project future increases of summer continental interior drying and associated risk of droughts. The greater overall risk is ascribed to further increases in temperature and resultant potential evapotranspiration not being offset by precipitation increases [3]. In fact, the recent Fifth Assessment Report from the IPCC [29] stated that an increase in the intensity and duration of future droughts on a regional to global scale is likely (*i.e.*, “medium” confidence) by the end of the 21st century. This statement especially applies to southern Europe and the Mediterranean region, central Europe, central North America, Central America and Mexico, northeast Brazil, and southern Africa. Elsewhere there is overall “low” confidence because of inconsistent projections of drought changes (dependent both on the GCM model and dryness index). Definitional issues, lack of observational data, and the inability of models to include all the factors that influence droughts preclude stronger confidence than “medium” in drought projections (see [30]).

Some global studies have indicated that droughts will be slightly more frequent and longer in duration as compared to present-day conditions by the second half of the 21st century [31,32]. Specifically for North America, Trenberth [33], using several GCM simulations, showed that future warming will increase both evaporation and surface drying, thereby leading to longer and more intense drought particularly in the summer over continental interior regions of the continent. A recent study found that the southwest and central plains regions in the continental U.S. will be significantly drier by the end of the century when compared with historical periods. This was the case even across various GCMs, and different metrics of soil moisture in both moderate and high greenhouse gas emission scenarios [34]. 

Overall, future precipitation/water resource projections suggest that in Canada, the North will become wetter and the South drier especially in summer [35]. However, limited research has been carried out regarding potential future drought occurrence across Canada and most investigations have focused on the particularly drought-prone Canadian Prairies. A first-order assessment of future drought occurrence over southern Canada (2041–2070) by Bonsal and Regier [14] found future annual precipitation will increase over most of the southern region of Canada but this will be more than offset by increased temperatures that lead to higher evaporation so increases in drought are likely. The future mean PDSI estimates indicate the prevalence of more frequent drought for the southern half of the Canadian Prairies in the latter half of the 21stcentury with persistent negative PDSI values expected after 2040 [15]. This suggests the possibility of a regime shift to a more arid climate in the southern Prairies due to the effects of projected summer temperature increases. Future projections of PDSI also revealed that multi-year droughts lasting 10 or more years are more likely than what has been historically observed. Projections suggest approximately three such events per 100 years as compared to the approximately once every 100 years that has been observed. Similar results for the southern Canadian Prairies were also documented by PaiMazumder *et al.* [36] using simulations from the Canadian Regional Climate Model for the period 2041–2070. Although published studies for the rest of Canada are limited, the authors noted that in more northern areas of the Prairies, drought events are projected to be less severe and less frequent. Considerable uncertainty exists with respect to future precipitation events, particularly on a regional and intra-seasonal basis. Bonsal *et al.* [15] found considerable range in projections in their assessment of changes to the temporal and spatial characteristics of future (2011–2100) PDSI and SPI using statistically downscaled daily temperature and precipitation data over the southern Canadian Prairies, from three GCMs with different greenhouse gas emission scenarios.

In summary, although there are no comprehensive analyses of future drought events across all of Canada to provide detailed regional and seasonal drought information, both global investigations and a handful of studies conducted for the Canadian Prairies suggest that future droughts will likely increase, particularly in those regions that already experience these events (*i.e.*, more southern and interior areas of the country).

## 2. Experimental Section

Canadian and U.S. literature was searched using PubMed, SCOPUS and Cochrane Collection databases. The following search terms were included: drought, arid * (* = the wildcard search character), desiccation, dry spell, water AND (shortage* OR scarcity), (low OR drop OR reduc *) AND (water AND (level OR table)), Canada, Canadian, health. Articles published over the 20 years from 1993 to 2013 were considered for the initial search. Articles were screened by scanning titles and/or abstracts. Full articles were scanned where abstracts were missing or unclear. Articles were included where the linkages to human health and drought were made explicit. Articles from reference lists and/or in-text citations meeting these criteria, as well as from climate change and vector-borne disease expert-provided sources (peer-reviewed and grey literature) were also included.

The international literature was searched using the OVID Medline and Web of Knowledge databases. The following search terms were included: climate, climate change, health, public health, drought, adaptation, response. Articles published at any time up to December 2013 were considered. Relevant websites, such as the World Health Organization, IPCC, United Nations Framework Convention on Climate Change were also searched using the same key words. Where appropriate, the entire article was reviewed for more detailed information. Articles were included if they discussed drought and human health effects and made explicit the link between the two, or if they discussed potential adaptations or responses to drought conditions. They were also included if the study was not limited to the examination of drought in a developing country/region and/or the results were applicable to the Canadian context. A number of articles were excluded given their limited relevance to the Canadian context including those focused on malnutrition, or infectious diseases that are rare in Canada, such as malaria, chikungunya, schistosoma and tick-borne relapsing fever [37,38,39,40]. Vector-borne disease expert articles were also included. The review of the Canadian and U.S. literature identified 64 records and the international literature yielded 72 records (duplicates were removed). This total (136) included 22 publications identified by climate change or vector-borne disease experts and 59 records identified within the in-text citations and references from the sources mentioned above. Broadly, evidence from developed countries and particularly from the U.S., Australia and the UK was considered to have greater potential relevancy than research focused on developing countries for this review, given their greater similarity to the Canadian context and of their populations in aspects that could modify health impacts related to drought (e.g., lifestyle, infrastructure and public services).

## 3. Results

### 3.1. Health Risks Associated with Drought

**Figure 1:**
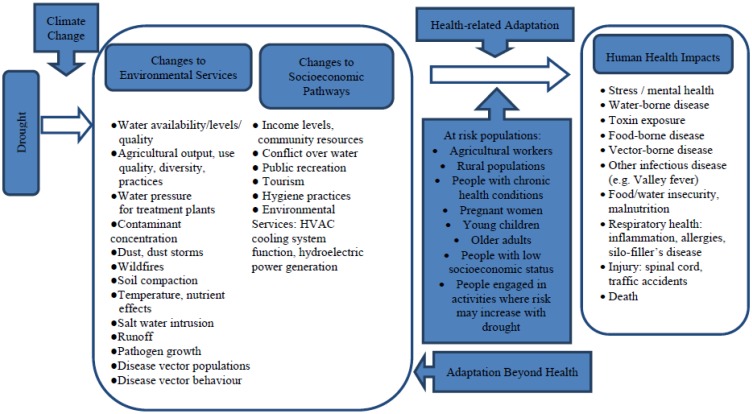
Pathways through which drought impacts human health in the context of climate change.

Drought can affect human health through a broad range of pathways including those shown in Figure 1. They are discussed in greater detail in the following sections. Generally speaking, these broadly fall into environmental services and socioeconomic areas. There is agreement that globally, drought leads to an increase in morbidity and mortality [41,42,43,44]. Although discussions have pointed to direct drought-related deaths in low-income countries [45], a broad range of public health impacts associated with drought has been identified in the U.S. [46,47,48]. Research also indicates that climate change may impact drought-related health outcomes in Canada [2,49,50]. In fact, some evidence of drought-related health impacts has been identified for Canadians living on the Prairies [51]. For example, there is some evidence of respiratory impacts related to dust and increased risk of water-borne disease [51]. Furthermore, reviews of impacts from Ontario [52] and the U.S. [53] have identified climate change and future drought as having the potential to affect food-, vector- and water-borne diseases [54,55].

### 3.2. Water Quality and Human Health

#### 3.2.1. Source Water for Drinking Water

Drought can negatively impact the quality of drinking water [2,23,46,50]. Decreases in drinking water quality can, in turn, lead to water-related disease [42,56,57,58,59,60] and the exacerbation of heat stress [61]. The pattern of reduced water levels, stream flow and resultant stagnation during drought can increase contaminant concentrations in ground and surface waters [46,51,62,63]. For example, measurements taken during a drought in 2003 over the Rhine and Meuse rivers in Europe showed not only a substantial reduction in river discharge relative to the year before, but also that chloride concentrations were inversely related to the flow (m^3^/s) of these rivers [64]. This effect is thought to increase when contaminants continue to be added to the system [64]. Lower water levels can also facilitate an increase in reactions between the water, contaminants and sediments [62]. This can be further reinforced by greater soil erosion during drought [23]. In the U.S., elevated nitrate, orthophosphates, chlorides and sulfates in groundwater have also been associated with drought, in some cases exceeding the U.S. EPA’s Maximum Contaminant Level [63]. Frequent detection in wells near agriculture and septic systems suggested these systems were at risk of contamination [63]. Baures *et al.* [65] found that variation in total organic carbon and nitrate was associated with changes to river flow in France, with a high ratio of total organic carbon to nitrate concentrations under conditions of very low river flow. A UK study found that drought can lead to greater contamination of drinking water source water from dissolved organic carbons and can increase difficulty in contaminant removal [66]. Similar patterns have been reported in Canada. Elevated nutrient concentrations (*i.e.*, phosphorous and nitrogen) have been observed in association with low water periods in Alberta [67].

Nutrient-loading is known to lead to eutrophication and is associated with cyanobacterial (algal) bloom development in all provinces [68]. Some cyanobacteria produce toxins that are harmful to humans and blooms can impact water bodies used as sources of drinking water and for recreational water activities [2]. In Nebraska (U.S.), for example, drought was considered a contributing factor to a cyanobacteria bloom in 2004 that resulted in reports to public health of impacts such as gastrointestinal illness and the issuance of health alerts and advisories for nearly 100 lakes [69]. However, Barbeau *et al.* [70] carried out a hypothetical analysis of the vulnerability of Quebec’s provincial drinking water systems to toxic cyanobacteria and their results suggest that existing systems in that province would be able to cope with this risk, even within the context of climate change. In Ontario, there was a significant increase in algal blooms between 1994 and 2009 [71]. Climate change may lead to changes in the frequency and duration of algal blooms, as well as increases in the amount of toxins they produce or an increase in the prevalence of toxin-producing species overall in temperate regions [70,71]. Warmer temperatures anticipated with climate change may promote algal blooms through mechanisms such as reducing the mixing of water, allowing algae to rise to the surface more quickly and by increasing the salinity of freshwater bodies [72].

The warmer temperatures associated with drought and decreases in water flow and/or volume may promote the survival and growth of pathogens in water, including those responsible for infectious disease [46,55,73]. Reduced water flow can lead to an increase in bacterial concentration and reduced water quality as there is less water available to dilute these contaminants [64,74].

Climate change-related impacts on water, including drought, are recognized as leading to greater risks from water-borne diseases such as hepatitis A [52]. However, there appears to be limited discussion of the relationship between drought and water-borne pathogens and the interactions between climate change and water-borne diseases are complex. In the U.S., the survival and multiplication of bacteria leading to leptospirosis outbreaks may have been altered by temperature and pH changes resulting from the stagnation associated with drought [75]. Similarly, although *Naegleria fowleri* is rare, increased water temperature during drought can allow for the amoeba to grow in greater concentrations [46]. Drought conditions may concentrate pathogens while at the same time inactivate them (e.g., *Cryptosporidium* oocysts) [54]. In Canada, it is important to note that the greatest burden from enteric, food and water-borne diseases, drawing on 2012 data, is associated with *campylobacteriosis* (10,174 cases), followed by salmonellosis (6828 cases) and giardiasis (3862 cases). Other reportable diseases in this category have a far smaller number of cases, such as shigellosis with 1,068 reported cases and approximately 500 cases each of *E. coli* and cryptosporidiosis in 2012 [76].In addition, while at its peak in 1970, there were 12,283 cases of hepatitis A reported in Canada; this has since fallen to approximately 200–300 cases per year [76]. Future changes to the frequency and/or severity of drought could modify the burden associated with these diseases in Canada.

In coastal areas, drought can result in the contamination of groundwater drinking water sources by the intrusion of salt water [46,50]. The mingling of saline and fresh groundwater that occurs in such regions can progress inland affecting freshwater wells, under a number of circumstances, including drier periods [77]. Saltwater intrusion can be exacerbated by climate change and sea-level rise [50,77].

The combination of drought with extreme precipitation may also impact water-, food- and vector- borne diseases. The most commonly cited impact of climate change on water-borne disease is the anticipated effects of increasing frequencies and amounts of heavy rainfall impacting the likelihood that untreated and pathogen-carrying water is consumed by Canadians [52]. Drought can amplify the impacts of a subsequent extreme rainfall event. During a drought event the soil can become compacted, thereby increasing runoff and the likelihood of water contamination and water-borne illness from precipitation events that follow [46,54,55,78]. Conditions associated with drought, such as low rainfall, can also concentrate *Giardia* and *Cryptosporidium* cysts in groundwater sources and where water is stored. Water sources may then be contaminated by the cysts after rainfall events [79]. Evidence from the Prairies suggests that the prevalence of livestock farming may affect water-borne diseases that follow such events if manure is able to contaminate water sources (e.g., *Eschericia (E). coli*, *Cryptosporidium*, *Giardia*) [80,81,82,83]. *Campylobacteriosis* outbreaks are also frequently associated with heavy rainfall, often when they follow a period of drought [54]. Although a much smaller risk for Canadians, with typically only 0–2 cases in Canada each year [76], the pattern of drought conditions followed by a wet spring could also activate anthrax [52].

#### 3.2.2. Treated Drinking Water

During drought, there can be a drop in pressure at water treatment plants [52], which can increase turbidity leading to a greater potential risk of water contamination and consequently a greater need for water treatment [84,85]. The impact of drought on contaminant levels in source water has been discussed above. In the event that well water becomes unavailable during drought, turning to alternative water sources can also increase risks to health if appropriate safeguards for those treatment measures are not in place [86]. Inadequate water treatment is important as pathogens, including bacteria and viruses, are known to be taken up by particles in water [87]. Costs also increase with the additional treatment required to address higher levels of turbidity [88,89]. Changes to water flow and levels associated with climate change could further contribute to stress on water treatment systems and increase the cost of treatment [50]. In northern Ontario, some First Nations communities may already be near the limit of their capacity in this regard [50].

#### 3.2.3. Recreational Water

Drought can also affect the quantity and quality of recreational water [59,60,61,65,90,91,92,93,94]. The hot and dry weather that can be associated with drought is thought to increase recreational water use [46,75]. At the same time, the increased concentration of pathogens encouraged under these conditions, as described above, are thought to increase the likelihood of infection among recreational water users [46]. Although leptospirosis cases and outbreaks are frequently associated with heavy rainfall events [57], in the U.S., they have also been associated with drought and with swimming in lakes [75]. In Canada, even under typical (non-drought) conditions, acute gastrointestinal illness (AGI) is a risk for swimmers, with the risk of AGI at approximately 3%–8% [95]. Although the ingestion of water during recreational use would be lower than that from the drinking water sources discussed above, recreational water users could be exposed to water-borne pathogens that lead to infection through direct contact or inhalation [96,97].

### 3.3. Sanitation/Hygiene

Water plays a key role in maintaining hygiene, which is directly associated with preventing disease [46,60,98]. Health care facilities may depend heavily on water to protect the health of patients and workers [46]. Studies outside of Canada suggest strong linkages between changes in precipitation, drought and diarrheal disease [99,100]. The World Health Organization has estimated that globally, the risk factor “water, sanitation and hygiene” accounted for 4% of all deaths [101]. A global cross-sectional study found the incidence of diarrhoea in children under the age of five years increased 4% with each 10mm per month decrease in precipitation [102]. Modeling done on a global scale by Motoshita *et al.* [103] based on recent historical data suggested that health damage from infectious diseases resulting from domestic water scarcity would be relatively low in the U.S. compared with other regions, although the health damage could not be modeled for Canada. However, in England and Wales, both low and excessive rainfall over the short-term was linked to a rise in the incidence of diarrhoea related to *Giardia*, *Cryptosporidium*, *E.coli*, *S. typhi*, *S. paratyphi*, *Campylobacter* and *Streptobacillus moniliformis* (This is not a reportable disease in Canada and infections are thought to be lower in Canada and other Western countries than in the US; close to 200 cases of “rat bite fever” from *Streptobacillus moniliformis* had been documented in the U.S. at the time of a 2007 publication [333]) [104]. Furthermore, people displaced by drought are considered likely to experience loss of sanitation and routine hygiene [45]. Human behavior may play a role, since the relaxing of hygiene practices could lead to an increase in diarrheal illnesses during drought [74]. As described above, *Giardia*, *Cryptosporidium*, *E. coli* and *Salmonella* cause a considerable number of disease cases in Canada each year. Alteration of the rates of such diseases by drought could have implications for public health.

### 3.4. Food-borne Diseases

The conditions associated with drought could impact food-borne illness. Hot and dry conditions often associated with drought are thought to favour the proliferation of some food-borne pathogens. The impact in Canada could be significant, as currently there are an estimated 11 million cases of food-borne illness annually across the country [105]. Among reportable diseases, 10,174 cases of *campylobacteriosis* and 6,828 cases of salmonellosis were reported in Canada in 2012 [76]. Illness caused by *Clostridium perfringens* is not nationally notifiable in Canada, but ranks among the most common food-borne diseases in the industrialized world [106]; in the U.S., *C. perfringens* is thought to cause close to one million cases of food-borne illness each year [107].

In some cases the environmental conditions created by drought are favorable to pathogens, such as *C. perfringens*, which does well in hot and dry conditions [52]. *Campylobacterios* is also expected to benefit from some seasonal conditions under climate change in the future [54]. However, the expected impacts of climate change-related drought conditions remain uncertain for other pathogens, including *Salmonella*, *Listeria* and *Norovirus* [54]. For example, under experimental desiccation *Salmonella (typhimurium)* lost infectivity and cultivability [108]. Meanwhile, in contrast to earlier studies, Zhang *et al.* [109], in comparing models to forecast salmonellosis cases in Adelaide (Australia) found decreased rainfall was associated with salmonellosis cases. It is possible that this finding was due to interaction with other variables such as temperature or the unique local climate [109]. For example, Ge *et al.* [110] suggest that under extreme conditions, including drought, when the soil contains high concentrations of *Salmonella* there may be greater internalization of the pathogen in lettuce relative to optimal irrigation conditions.

During drought, cultivated crops, fish and shellfish consumed for food [52] may pose an increased risk of disease, which could have implications for human health [46]. If treated municipal sewage is used by the agricultural sector for irrigation or to process products due to water shortages there can be an increase in the risk of food-borne illness (e.g., *Salmonella*, *E. coli*) [46]. Local food production practices and food imported from some countries may need to be monitored more closely under such conditions [46].

### 3.5. Vector-Borne Diseases

#### 3.5.1. West Nile and St. Louis Encephalitis

Vector-borne diseases—particularly mosquito-borne ones—are frequently associated with increased precipitation [111] because standing water is needed for the development of immature stages of mosquitoes. Mosquitoes are important disease vectors in Canada as they can transmit West Nile virus (WNV). There were 110 total clinical cases of West Nile virus reported in Canada between January and the end of November, 2013, including three cases potentially related to travel outside the province where they were reported [112]. High precipitation in the prairie provinces in 2007 was thought responsible for an unprecedented abundance of *Culex tarsalis* mosquitoes that in turn led, later that year, to the largest ever outbreak of WNV in Canada (2,401 cases) [76,113,114]. Studies on the mosquitoes *Cx. pipiens* and *Cx. restuans*, which are the main WNV vectors in eastern Canada, also suggest that precipitation is positively associated with their abundance [115]. However, in some circumstances periods of drought have been directly associated with increased risk from mosquito-borne infections, such as WNV in southeastern, northeastern, central and western U.S., the Canadian prairies, as well as in Europe [115,116,117,118,119,120,121,122]. A number of explanations have been proposed for these observations, including impacts of drought on competitors and predators of WNV mosquito vectors [116] which can lead to mosquito population increases [115] and changes in communities and densities of the bird species that are reservoir hosts of WNV, resulting in increased WNV prevalence in mosquitoes [123,124]. Semi-permanent rather than permanent wetlands are thought to be particularly prone to the effects of drought given the fluctuations they experience in response to precipitation. Irrigation during times of drought may create new breeding sites for WNV mosquito vectors and thus shift the local geographic focus of risk from WNV [125]. In the matrix of mosquito breeding habitat found in urban and suburban environments, small numbers of locations can contribute to mosquito breeding and abundance and these are frequently man-made water courses, sewage overflow systems and holding pools, *etc .* [126], which are likely to continue to hold water during drought events. Associations of WNV incidence with droughts or low precipitation (e.g., in Virginia, U.S. [127]) may be due to reduced flushing of mosquito larvae and eggs from breeding sites in urban areas during rainfall; this flushing reduces mosquito abundance [128].

Frequently, the effect of drought on vector-borne diseases is seen when drought is followed by periods of wetter weather [129,130], but the relationships amongst mosquito abundance, infection prevalence, WNV risk and seasonal variations in drought and rainfall can be complex, geographically variable, habitat/environment-specific and difficult to predict [131]. In addition to altering the mosquito vectors’ environment, drought can impact risk from mosquito-borne diseases by affecting contact rates between the vectors and reservoir hosts, thereby influencing the prevalence of infection in mosquitoes [46,118]. Evidence for this mechanism comes particularly from studies on mosquito-bird transmitted viruses [118] including WNV [129] and St. Louis encephalitis (SLE) virus [132].The congregation of reservoir host birds around dwindling water sources may be one way that drought affects WNV infection prevalence in mosquitoes by increasing rates of contact between avian reservoirs and mosquitoes thereby amplifying transmission [129,132]. In California (U.S.) drought may have contributed to an increase in human WNV cases by reducing populations of non-competent host birds, allowing for greater contact of mosquitoes with reservoir-competent host birds and possibly humans [131]. In the southeastern U.S., SLE outbreaks are associated with rainfall following drought, which is largely due to the behavior of the main vector *Culex nigripalpis* [133]. In drought conditions, gravid *Culex nigripalpis* females will increase in numbers around standing water as they wait for rainfall to feed and lay their eggs together, resulting in synchronous production of mosquitoes [134]. In addition, enhanced transmission due to the concentrating of mosquitoes and bird reservoirs in habitats that hold water is thought to help amplify the SLE virus [132,135].

#### 3.5.2. Lyme Disease

Lyme disease, the most frequent vector-borne disease diagnosed in North America, is emerging in Canada [136,137]. Drought has a consistently negative effect on risk from tick-borne diseases such as Lyme disease because of (i) direct killing of ticks by desiccation in severe drought; (ii) reduction in host-finding success by reducing the time that ticks can spend seeking hosts outside of moisture-holding refugia in the habitats in which they occur; and (iii) increased energy expenditure due to ticks returning more frequently to moisture-holding refugia to rehydrate [138,139].

#### 3.5.3. Directly Transmitted Wildlife-borne Zoonoses

The effect of drought on zoonoses transmitted directly from wildlife to humans is likely a reduction in the abundance of wild animal hosts and consequently, reduced pathogen transmission. When drought is followed by rainfall, wild animal host densities may increase again, but as most individuals will be naïve of infection, the potential for epidemics exists. Rodent-borne hantavirus is a paradigm for this in which drought followed by heavy rainfall (particularly associated with El Nino) is thought to drive epidemics in the southwestern U.S. [53,140,141].

### 3.6. Fungal Diseases

Drought may encourage the growth of various fungi associated with health impacts. Warmer, drier summers may have contributed to the establishment of *Cryptococcus gattii* in Canada [142]. Low moisture has been associated with *C. gattii* soil colonization [143]. The fungus was first detected on Vancouver Island in 1999, with more than 100 human cases identified [142]. Between 1999 and 2007, there were 218 reported cases of *C. gattii* in British Columbia, the region with the largest *C. gattii* infected population in Canada and the world [144].

Drought may also positively affect the growth and dissemination of *coccidioidomycosis* (Valley fever). Rainfall patterns and timing (including length of the drought) have been identified as modifiers of the incidence of this disease [46,142,145,146,147,148]. Although the fungus is concentrated in the Southwestern U.S., recent cases that appeared to originate in Washington State (U.S.) suggest that it may be capable of moving to other areas [149]. Valley fever is presently rare in Canada but climate change may impact the incidence of the disease among Canadian travelers [150]. Since 1952, there have been a total of 128 cases of *coccidiodomycosis* in Canada. These Canadian cases were acquired during travel outside Canada [150].

Although associated with warming rather than drought, projections of increased precipitation in the winter months followed by reduced precipitation in the summer months for North America are thought to favor the dispersal of *Blastomyces dermatitidis* spores, which causes *blastomycosis* [142]. Currently, this fungus occurs mainly in northwestern Ontario [142]. Between 1994 and 2003, there were 309 cases of *blastomycosis* in Ontario with more than half of the cases occurring between 2001 and 2003 [151]. Geographically, northern Ontario accounted for 61% of the cases, followed by 21% from the Toronto region [151].

### 3.7. Respiratory Health

#### 3.7.1. Dust, Particulates and Allergens

Drought is often accompanied by dry, dusty conditions and dust storms, which can impact health [83,152,153]. Climate change can lead to an increase in fine particulate matter, allergen and dust concentrations in the air in drought-prone areas [154] which can have significant health impacts. A study of a lake desiccated through persistent drought in Saskatchewan found a greater prevalence of coughing, wheezing and eye and nasal irritation among residents living near the lake than among those in a control group [155]. PM_10_ (air particle) increases during and following dust storms have also been found to cause small increases in emergency room visits for bronchitis and sinusitis in the U.S. [156] although dust storms were not associated with an increased risk of mortality [157]. Air-borne toxins from algal blooms, which can be increased due to drought conditions, can also irritate the respiratory system, nose and eyes [46,153,155]. It has been anecdotally suggested that there were numerous deaths from “dust pneumonia” related to dust inhalation during the drought of the 1930s in the U.S. [152]. In Ontario, Charron *et al.* [52] noted the possibility that drought with strong winds could lead to the transmission of Rickettsial diseases to humans from infected livestock.

#### 3.7.2. Silo-Filler’s Disease

Dry conditions can increase nitrate levels in corn plants and encourage the accumulation of nitrogen dioxide (NO_2_), thereby elevating the risk of “silo-filler’s disease” [158]. A greater number of “silo-filler’s disease” cases were reported in New York State after a dry growing season in 1995 [158]. There are approximately five cases of silo-filler’s disease per 100,000 silo-associated farm workers in New York State annually [159].

#### 3.7.3. Wildfires and Extreme Heat

Drought has been associated with wildfires in both Canada and the U.S. and is expected to increase the number of wildfires in the Prairies specifically [46,51,83]. The international literature links wildfires to respiratory, cardiovascular, ophthalmic and psychiatric illnesses [43,160,161]. In Brazil, a study of young children linked an increase in respiratory disease incidence with drought-related wildfires [162]. During a prolonged forest fire in British Columbia, Canada in 2003 one community experienced a 46%–78% increase in physician visits for respiratory diseases [163]. Wildfires and other extreme weather events that might lead to displacement can be a source of significant stress for those directly affected [51,80,83,160,164]. The significant loss of property and infrastructure during a wildfire can also have negative impacts on mental health [51] and may lead to post-traumatic stress disorder [160,164].

More recently, in 2011 the northern Prairies experienced severe drought and catastrophic fires during which Slave Lake, Alberta lost one-third of homes and businesses to wildfire [165,166].In the Northwest Territories, a dry winter combined with only half the typical amount of rainfall over the summer of 2014 and above average temperatures, was accompanied by the worst fire season in 30 years [167]. Record warm and dry conditions in British Columbia over the same summer and preceding winter also set the stage for a wildfire season that saw more than seven and a half times the 20 year average in land area burned in 2014 [167]. Wildfires are expected to become more frequent and severe in many parts of Canada with climate change [168]. Further, drought has been associated with periods of extreme heat which can present a range of health impacts that need to be addressed. In addition to its direct impact on human health, extreme heat can add to drought-related stress on agriculture with knock-on effects discussed below. For example, an extreme heat event that took place in Russia over July and August, 2010 reduced the country’s grain yield by 25%, resulting in increases in food prices and losses estimated at $15 billion [169].

### 3.8. Injuries

Although severe injuries directly resulting from drought may be unlikely in mid and higher income countries [45], during periods of drought an increased incidence of spinal cord injuries has been recorded in the U.S. from diving into shallow water bodies. Accidents from debris can also be a concern as lower water levels can expose previously “hidden” hazards in bodies of water [46,170,171]. Affected individuals may have been seeking relief from warm temperatures accompanying dry conditions [171]. Although carelessness and alcohol may play a role, injured individuals were often familiar with local bodies of water and believed the water to be deeper than it actually was [170,171]. Dust storms, discussed above in the context of drought, have also been associated with traffic accidents on the Prairies [23,153,172]. Dust storms can reduce visibility for drivers [172].

### 3.9. Food/Water Insecurity

Drought may increase the difficulty that individuals face in obtaining adequate water. In the U.S. there have been reports of risk of drinking water shortages [173]. In Canada, there have been reports of costly water-related infrastructure such as water cisterns, septic tanks and wells damaged or at risk during drought [80,174,175,176].

The IPCC recognizes a broad range of impacts due to climate change and the altered frequencies of associated extreme events, such as drought and floods that can have negative effects on crop production [177,178,179]. Climate change can also alter the geographic distribution and productivity of crops and livestock and the distribution of pests and disease [180,181,182]. Food security can be affected through drought-related decreases in agricultural output, food shortages and higher food prices. Crop failures and reduced crop yields are of particular concern because they can have immediate and direct effects on both local and global food security [183,184,185,186,187].Climate change could lead to a decrease in the zinc and iron content of staple crops such as wheat [188]. Such a change in nutritional content could exacerbate iron deficiency, which occurs in both developed and developing countries [189].

Meanwhile, increasing homogeneity among crop species globally threatens food security with increased interdependence of food systems around the world and reduced genetic diversity [190].As a net exporter of food and the 9th largest exporter in the world [191], Canada is unlikely to experience the types of food shortages that could occur in other countries. However, food insecurity may be reflected in food price volatility caused by international market shortages [192], which could impact low income Canadians who find it difficult to pay higher prices for nutritious food. In addition, some individuals may be forced to use their resources to purchase water instead of food [46,49]. The media reported a surge in food bank users and pointed to high household spending on water in parts of California (U.S.) during their recent drought [193]. The widespread U.S. drought in 2012 was thought to be one of the factors contributing to an increase in food prices in countries, including Canada [194,195].In addition, threats from climate change to the food security of First Nations and Inuit populations in northern Canada are increasing [2]. While it appears to be complex, a recent Australian study suggests that drought may play a moderating role in the relationship between food insecurity and psychological distress, possibly increasing distress [196].

For those who rely on subsistence fishing, such as Aboriginal populations, health can be affected when drought affects the health of aquatic organisms [46]. Drought can increase the likelihood of disease spread and/or exposure to an accumulation of contaminants in the water for these aquatic organisms [46]. They can also be impacted by decreases in the quality and quantity of water during drought or through contamination introduced by the rains that follow drought [52].

### 3.10. Socioeconomic Pathways Leading to Human Health Impacts

Drought can affect social and economic wellbeing within a community through changes to water availability that can impact agriculture [176]. The Canadian agricultural sector produced crop and livestock products totaling over $41 billion in 2010 [197]. Costs associated with a drought can be significant. For example, total costs of drought across the Prairies in 1990 exceeded $800 million (equivalent 2011 Canadian dollars) [198]. The more recent drought of 2001–2002 was responsible for $3.6 billion in direct losses related to agricultural production [199]. Impacts on agriculture are passed on to the broader community and may lead to temporary unemployment [200] and increase stress levels [176,201]. The U.S. drought in the 1930s and its impact on agriculture contributed to the Great Depression; there was high unemployment, increased demands on government aid programs and community impacts as millions of people affected by the drought moved to other communities [202].

The impact of drought on different segments of a society can vary [203]. For example, the 2001–2002 drought brought significant impacts on agricultural production in Alberta’s “Special Areas”, but it was mainly an inconvenience for residents and businesses outside the agricultural sector [203]. Similarly, the 1987–1992 drought in California (U.S.) led to primarily behavioral rather than economic impacts for residents [200]. Loss of agricultural output in one area may drive up the demand and prices for farmers unaffected by the drought suggesting that some economic impacts may be highly localized [204]. Globally, the impact of climate change on agriculture in different regions and countries, such as other developed grain-exporting countries can affect the competitiveness of Canada’s agricultural sector, particularly for wheat [174]. A widespread U.S. drought in 2012 increased crop prices, resulting in new record highs for Canadian farm incomes overall [205].

The international literature suggests that drought can impact communities by shifting resources from services such as public health to address more immediate needs (e.g., sanitation), as well as leading to conflict over water between groups and regions [2,42,59,90,206,207,208]. Recent media attention around conflicts over water in the western U.S. states among farmers, activists, developers and governments at state and local levels suggests that conflict over water in developed countries such as Canada could be a concern [209]. During periods of drought, water-related recreational activities may be cancelled or closed, such as during the 2001–2002 drought in the Prairies and the 1987–1992 drought in the U.S., which can have social impacts [174,176,200].

In the Canadian context, data from 2005 show that over 90% of the water withdrawn in the country was to support economic activity [12]. The impact of drought on economic activities overall, in particular through reductions in power generation capacity, could be considerable. For example, between 1999 (winter) and 2004 (fall), seasonal decreases in precipitation of up to 60% compared to normal conditions were observed in the Canadian cities of Calgary, Edmonton and Saskatoon [9]. Hydroelectricity generation accounts for 62% of electricity generation in Canada [12] (A large amount of hydroelectricity is generated in Canada and when in-stream use is taken into account, this industry accounts for the largest amount of water use in Canada [12]. The volume of water used for hydroelectricity generation was over 70 times the total of all other combined water use in Canada in 2005 [12]. Thermal (nuclear and fossil fuel) power generation and manufacturing are responsible for the largest and second largest total withdrawals of freshwater in Canada [12,334]). Shortfalls in this area could further amplify costs and stresses associated with drought, for example through the lack of revenue or the need to purchase alternative energy (see section 3.12.2 for additional discussion of the health impacts of these shortfalls). During the 2001 drought, hydroelectric power generation in Saskatchewan fell to 66% of the past four year average in the province resulting in power being purchased from other jurisdictions to meet electricity needs [174]. During the 1987–1992 drought in California (U.S.), hydroelectricity generation fell to under 60% of normal levels [200]. Additional energy was purchased from more expensive sources [200] and led to more than a $3 billion increase in electricity costs for consumers [204]. Drought may exacerbate the substantial impacts anticipated with climate change. The climate change-related decrease in water levels anticipated across the Great Lakes is expected to result in direct economic impacts between 2014 and 2030 of $9.61 billion (2012 USD) due to impacts on recreation, shipping and harbours, hydroelectricity generation, waterfront property values and groundwater use [210].

### 3.11. Mental Health

**Figure 2:**
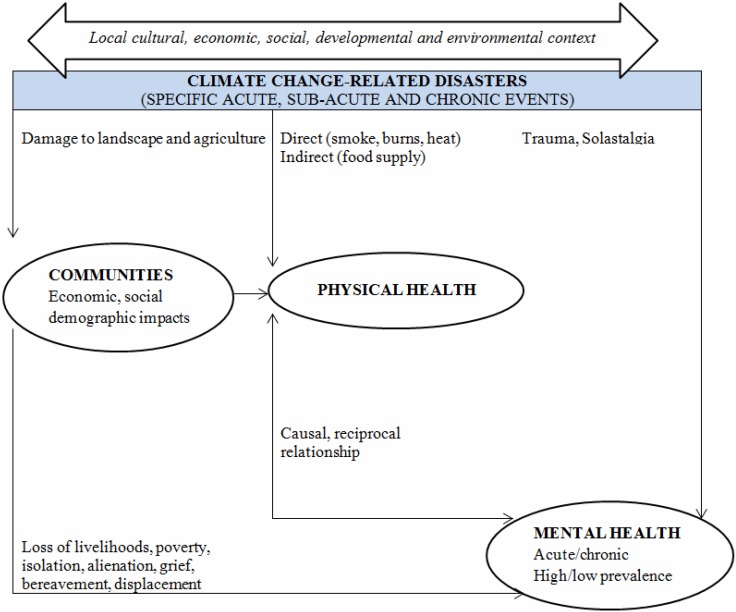
Pathways through which climate change-related disasters can affect mental health. Source: Reproduced from Berry *et al.* [220].

Drought and its associated financial burden can lead to stress [51,176,201,211,212,213,214] and is associated with physical and mental health problems, such as anxiety, emotional and psychological distress, loss and grief [46,215,216,217,218]. The pathways leading to these impacts are illustrated in Figure 2. For farm families, financial stress [51] and drought [219] can amplify the other stressors they experience. For consumers with low socio-economic status, stress related to an increase in food costs or the decreased availability of food can also negatively impact mental health [46].

Australia has produced numerous studies regarding drought’s relationship to mental health. Negative drought-related impacts on mental health have been identified for older women and Aboriginal groups in Australia [216,221], as well as for rural Australian youth [222], with emotional stress increasing with the length of the drought [217]. Dryland salinity, a phenomenon similar to drought, has also been associated with an increased risk of hospitalization for depression in Australia [223]. Although Stehlik *et al.* [214] found that drought-related stress negatively impacted relationships within farm families (Central Queensland, New South Wales, Australia), a later large-scale study across Australian agricultural communities did not find family relationships were negatively impacted by drought [224]. For drought that was both constant and long (although not for either alone), there was a difference in the distress levels in rural and urban populations in Australia, with relatively higher levels among rural populations [225]. The degradation of the physical environment associated with drying could also contribute to psychological distress for those who feel connected to the landscape (solastalgia) [226].

Research from New South Wales (Australia) found a statistically significant relationship between drought and suicide from 1964 to 2001 [227]. Limited studies suggest that drought is a contributing factor to suicide among men in rural areas in Australia [228,229]. However, Page and Fragar [230] found a greater rate of suicide between 1988 and 1997 among male farm managers and agricultural labourers than among the male Australian population or the rural population overall, independent of drought.

### 3.12. Populations Vulnerable to the Health Impacts of Drought

Populations at higher risk to the health impacts of climate change have been identified in the literature. They include infants and children, elderly people, the socially and economically disadvantaged, pregnant women, people who spend time outdoors and those with chronic diseases and compromised immune systems (Table 1) [1,59,231,232,233]. Overlap exists between populations vulnerable to the health impacts of drought and populations at risk of adverse impacts on health resulting from other extreme weather events, including those expected to increase in frequency and intensity with climate change [46,59]. For example, people of low-socioeconomic status have more difficulty taking protective actions during extreme heat events [234] and may also suffer greater impacts from drought [49]. As an important pathway for increased exposure to injuries, air pollution, food, water and vector-borne diseases as discussed above, drought is associated with many higher risk populations. The existence of common vulnerability factors to multiple climate-related vulnerabilities increases overall risk to public health, but also offers significant opportunities to take adaptive actions that convey benefits among a broad spectrum of risk areas, including drought. For example, Cheng and Berry [235] identify efforts to enhance social capital as a climate change adaptation that has multiple health co-benefits.

**Table 1:** Vulnerability to climate-sensitive health outcomes by subpopulation [233].

Groups with Increased Vulnerability	Climate-related Vulnerabilities
Infants and children	Heat stress, air pollution, water-borne/food-borne diseases, vector-borne diseases, malnutrition
Pregnant women	Heat stress, extreme weather events, water-borne/food-borne diseases, vector-borne diseases
Elderly people and people with chronic medical conditions	Heat stress, air pollution, extreme weather events, water-borne/food-borne diseases, vector-borne diseases
Impoverished/low socioeconomic status	Heat stress, air pollution, extreme weather events, water-borne/food-borne diseases, vector-borne diseases
Outdoor workers	Heat stress, air pollution, vector-borne diseases, ultraviolet light (UV) exposure

The following sections describe how specific populations may be at increased vulnerability to the health impacts of drought.

#### 3.12.1. Agricultural Workers and Rural Populations

Agricultural workers and others who rely on water and/or rainfall for their livelihoods are at greater risk of experiencing the mental health impacts of drought [46,51,83,174,176,212]. Over time new agricultural practices and technologies have increased agricultural output, but have also led to increased costs and vulnerability for farmers during drought [83]. Given their occupational environment, agricultural workers are at increased risk of “silo-filler’s disease” (NO_2_ gas poisoning within silos) [158]. Horticulturalists and people working for nurseries, garden supply stores and recreational facilities have also been identified as being at higher risk of negative health outcomes [46].

Research in Australia suggests that drought-related stress on agricultural workers can be widespread. A survey of rural farmers in New South Wales, Australia found that 71.8% of rural farmers reported stress due to drought [213]. Limited evidence suggests that drought is a contributing factor to suicide among men in rural areas [228,229]. In some studies, younger farmers were found to experience higher levels of stress than their older counterparts between 55–64 years but not their oldest counterparts aged 65–74 years [212,236].

The mental health impacts of drought can extend beyond farmers themselves to their families [237]. Rural Australian youth have experienced negative mental health effects during prolonged drought [222], with their emotional stress increasing with the length of the drought [217]. Older women and Aboriginals have also been identified as being more vulnerable to these impacts [216,221]. In general, rural and urban populations can experience different levels of drought-related distress. For drought that was both constant and long (although not for either alone) in Australia, distress levels in rural populations were observed to be higher relative to urban populations [225].

#### 3.12.2. People with Chronic Health Conditions, Pregnant Women, Young Children and Older Adults

People with chronic health conditions are at increased risk of health impacts associated with air quality compromised by drought including the effects of wildfires [46,47,49,83,154,155]. This group is also thought to be at greater risk of drought-related infectious diseases [49]. Pregnant and nursing women, dialysis patients and immune-compromised individuals are considered to be at increased risk from health impacts of drought and heat [47]. Pregnant women in their third trimester, diabetics and immunosuppressed individuals were thought to have increased risk of developing a disseminated case of Valley fever [238]. Young children and seniors are at increased risk from drought-related water-borne diseases [49]. In addition to the very young (particularly those who have received rotavirus vaccines) and the elderly, those who are immunocompromised are at greater risk of developing acute gastrointestinal illness from water-borne diseases [95]. Very young children and seniors overlap with those susceptible to heat as identified above and may also be at greater risk from decreases in water quality and availability [60]. Children under five years of age and adults over 50 years old are also at increased risk of developing a disseminated case of Valley fever [146]. Individuals under institutional care (e.g., in hospitals, nursing homes) who depend on electrical equipment may be impacted by hydroelectricity power shortages during a drought [46]. Drought can also lead to disruptions of heating and cooling systems that rely on water to function, and these disruptions may result in health impacts on individuals when it is not possible to maintain health-protective temperatures [46].

#### 3.12.3. People with Low Socioeconomic Status

People with low socioeconomic status are at greater risk of health impacts from climate change [59]. For example, families who are already facing challenges meeting their needs or who engage in subsistence fishing or farming may be at risk of food insecurity with drought [46,49]. In Canada, 8.3% of households were food insecure in 2011–2012, with 36.7% of households in Nunavut food insecure during this period [30].

#### 3.12.4. People Engaged in Activities Where Risk May Increase with Drought

Individuals who spend more time outdoors have increased exposure to certain drought-related risks including those for respiratory diseases resulting from exposures related to wildfires and increased dust (e.g., dust-borne Valley fever) [59,146]. Exposure to vector-borne diseases, such as WNV or Eastern Equine Encephalitis, that may be linked to drought is increased in people spending greater amounts of time outdoors for recreation and/or due to their occupation (e.g., agricultural workers, parks services), placing them at greater risk of contracting these diseases [47,76]. Although the overall risk is considered to be low, individuals with greater exposure to the droppings of infected rodents are at increased risk of contracting rodent-borne diseases (e.g., Hantavirus) [239].

Recreational water users are at greater risk of contracting water-borne diseases, especially from pathogens favored by warm, shallow waters that often accompany drought [46,49,75]. In addition to biological pathogens, they may also have greater exposure to chemical contaminants [46]. Spinal cord injuries related to diving into shallow waters largely occur in males between 15–25 years of age [170].

### 3.13. Adaptation to the Human Health Impacts of Drought (Direct)

A broad range of adaptation strategies are available that help reduce the health impacts of drought. They generally attempt to reduce the impacts directly or they modify the environmental services or socioeconomic pathways through which drought affects health (Table 2). However, there has been little evaluation of the effectiveness of many of these adaptations [183,240]. Limited knowledge of the effectiveness of current measures to reduce health risks from drought increases the possibility of “maladaptation” as communities and individuals prepare for climate change. Ensuring that adaptation strategies are developed with evidence-based information and through consultation with a wide range of partners mitigates the risk of maladaptation. 

**Table 2:** Examples of adaptations to reduce the health impacts of drought.

**Adaptations to the human health impacts**
Water quality and water treatment monitoring
Disease surveillance and monitoring
Air quality monitoring and warnings
Public education and outreach
Mental health prevention and awareness programs
**Adaptations to address changes to environmental services or socioeconomic pathways**
Mainstreaming climate change into source water protection planning
Enhancing water service delivery and watershed management
Water capture and storage
Increasing water use efficiency and water conservation
Modifying or adopting new agricultural practices, such as increasing reliance on crop irrigation
Introducing drought-resistant crops and diversifying crops and income sources
Using seasonal climate outlooks for crop planning
Developing watershed management and climate change adaptation plans
Insurance and other financial assistance programs
Irrigation and altering the timing and use of water

#### 3.13.1. Monitoring Water Quality and Water Treatment

In addition to monitoring indicators for drought itself [47] or drought-related drivers of health impacts [241], increased water quality testing and monitoring of drinking water and/or recreational water during droughts can help safeguard health [43,59,65,74,92]. For example, the province of Quebec has begun to adapt by increasing the monitoring of surface water and adopting more stringent standards for water quality surveillance. Water treatment is also an important adaptation to protect against water-borne disease [59] and boil-water advisories may be a supportive tool, although they are designed to address fecal and not algal or chemical contamination. During the 2001 drought within the Kanai Blood Indian Reserve in Alberta (Canada), there was an increase in the number of boil water advisories called to protect residents against potential contamination [174].

#### 3.13.2. Disease Surveillance and Monitoring

Surveillance and monitoring of diseases sensitive to drought such as those caused by *E. coli*, *C. perfringens* and WNV are important adaptations [43,52,53,56,74,94,242,243,244]. Surveillance and monitoring can help to direct the provision of medical services and the development of needed vaccines to reduce risks to health [59,74,94,242,243]. Modeling could play a role alongside surveillance [125]. Control of disease vectors may also be implemented [59]. Primary care physicians can be part of monitoring efforts and help identify and address cases of malnutrition during drought episodes [207].

#### 3.13.3. Air Quality Monitoring and Warnings

When drought threatens air quality, for example by increasing particulate matter, air quality monitoring and warnings for the public can help reduce health risks [51]. In over 60 communities and 10 provinces across Canada, the Air Quality Health Index (AQHI) provides individuals with information on the immediate health risks associated with local air quality [245]. The AQHI includes information on how to reduce exposure to air pollution that is tailored to the general public and to vulnerable populations (e.g., parents with children and infants, seniors and those with cardiovascular and respiratory diseases). It is regularly updated and accessible through the Internet [246]. However, it may be challenging to capture a quickly-developing event such as a forest fire smoke episode through the Index. Environment Canada informs the public of severe weather by providing special weather statements, advisories and watches [247]. In responding to wildfires in western Canada in 2014, Environment Canada issued alerts through wildfire smoke warnings and special air quality statements [248]. Provincial and community-level agencies further monitor events such as forest fires and provide information to those affected through a number of platforms, including websites and social media [249].

#### 3.13.4. Public Education and Outreach

Public education is a key adaptation to protect health from drought. Personal protection measures that can be promoted by public health officials can include reducing water- and vector-borne disease exposures [59], wearing respiratory masks on days when ambient air quality could impact health [51], and the promotion of hand washing [74]. Education for travelers to and from Canada can raise awareness about diseases such as malaria [80], or tuberculosis, which may be increasingly transmitted under social conditions created by drought in developing countries [52]. To prevent spinal cord injuries resulting from diving in shallow waters, some jurisdictions have implemented public education, including the posting of notices where diving is not safe [170]. For example, brain and spinal cord injury prevention programs have been carried out in Saskatchewan and Ontario (Canada) and in Washington State (U.S.) [250,251,252]. However, evaluations of these programs suggest that program delivery needs to be well designed and robust to be effective. Although the initiative in Saskatchewan was found to change knowledge among students, in the Toronto (Ontario) case only 16% of students in schools where the program video was distributed viewed the information [250]. The one time intervention in Washington State, where students attended a session which included a video, lecture and personal testimony around traumatic brain and spinal cord injury, had little impact on knowledge or behavior [252]. Similarly, evidence suggests that boil water advisories may not be adhered to by everyone. During the gastroenteritis outbreak in Walkerton, Ontario (Canada), some residents under the boil water advisory continued to use and occasionally drink the water without boiling it [85].

The link between water and hygienic practices should be considered when developing drought adaptation measures. Conservation should take into consideration on-farm agricultural practices, regional water management and treatment and allow for and encourage individual hygienic practices such as hand washing [46]. In the later example, homes and businesses could also promote both water conservation and hand washing by installing low-flow faucet aerators [46]. Education of health care providers can also benefit efforts to address drought impacts on health [53].

#### 3.13.5. Addressing Mental Health Impacts

Adaptive actions may be taken by individuals and professionals within primary care and community services settings to reduce the mental health impacts of drought. Personal hopefulness and positive attitudes towards the rural lifestyle have been highlighted as useful adaptations to drought [218,253]. According to Caldwell and Boyd [254] rural Australian families used a combination of both positive (e.g., problem-focused coping, optimism and positive appraisal) and less beneficial strategies (e.g., cognitive dissonance, denial and avoidance of negative social influences) in their response to drought. Primary care practitioners can provide support to reduce symptoms of mental illness exacerbated by drought and ineffective coping mechanisms such as substance abuse. They may also provide information about financial support resources or community drought support groups [207,255]. Mental health programs in schools and in occupational settings and suicide prevention and awareness programs have also been identified as possible adaptation measures [59,216,222,256,257]. Newspapers may be another pathway to communicate broadly about mental health during drought [258].In Australia rural support workers help traditional mental health professionals identify farmers experiencing mental distress and refer them to support services [259]. Training in mental health first aid can also increase the capacity of rural support workers to address mental health needs in drought-affected rural areas [260]. One study suggested that the Rural Adversity Mental Health Program (RAMHP), which is based on a community development model, is effective in building capacity and resiliency to cope with the impacts of drought in rural New South Wales (Australia) [261]. Linking the services and programs focused on mental health, community or social needs with those on practical assistance for farming communities is considered key for addressing mental health concerns associated with or exacerbated by drought [262]. Access to support services for older individuals is another consideration [256].

Community-building [263] and events which increase social capital can further help decrease psychological stress [254]. High levels of social capital in many Canadian (Prairie) rural communities [176,264], based upon high levels of trust, networking and participation in organizations help reduce stress associated with drought [176,264]. However, Australian farm families faced heavier workloads during the drought of 2002–2003, which left less time for community participation, volunteer activities and hence could negatively impact social capital and the community [201].

### 3.14. Adaptation to Address Changes to Environmental Services or Socioeconomic Pathways (Indirect)

#### 3.14.1. Adaptation in Urban Environments

Effective adaptation to climate change-related impacts, including drought, requires “mainstreaming” in which climate change information is routinely considered and incorporated into existing programs, policies and planning [2,62]. For example, in Ontario, de Loe and Berg [62] point to opportunities to “mainstream” climate change into source water protection planning within the Clean Water Act to help address anticipated changes to water supply and demand, including an increased frequency of drought [62]. Mainstreaming can be assisted by integrating learnings about effective risk management options and adaptations from experiences with current climate variability, particularly extreme weather events. However, mainstreaming also requires consideration and use of information about risks expected from future climate change in adaptation planning. A range of measures to adapt to changes in water quality and quantity in the context of climate change, such as leak detection, outreach, adjustments to the building code and management policies have been reported in the province of Quebec [49].

Many sectors will be affected by drought and play a role in adaptation to reduce risks to individuals and communities. In urban centers communities may adapt to drought by enhancing water delivery systems and through watershed management [59,74,204]. In Atlantic Canada, communities facing shortages or reduced water quality have made efforts to improve water treatment systems, conserve and protect surface water and develop new water sources through, for example, water capture [50]. Dams, desalination and water recycling are also being used to cope with the increased variability in water supply, although the cost and impacts of such actions are not well established [204]. The health sector must actively be engaged in drought management at all levels of government. For example, in Brazil actions to protect public health are embedded into all stages of drought-related disaster risk reduction [241].

Increasing water use efficiency is advocated as an important adaptation when water infrastructure already exists [204]. Water conservation has also been highlighted as an important measure for easing drought-related costs [50,51,204]. This may be done through voluntary or mandatory restrictions on water usage during vulnerable periods [43] or education campaigns to promote conservation [50,51]. During the 1987–1988 drought in California (U.S.) education was used in conjunction with mandatory and voluntary water conservation programs to target a 10%–25% reduction in water use [265]. As this drought stretched on, impacts spread from dryland agricultural areas to impacts on state level electrical power supply. Lessons learned in a study of this drought identified inadequacies in water resource planning, the existence of responsible agencies along political rather than affected area boundaries and a lack of inter-jurisdictional communication as key factors. Water shortages continue to occur in California despite national freshwater draws (per capita) having dropped below levels seen in 1975 [265], with the exception of municipal and industrial use which are also expected to decline as regulations and technologies for more efficient use are adopted. In the most recent 2014 drought in California the entire state was under moderate to exceptional drought and water customers in Santa Cruz were required to decrease their water consumption by 25% with financial penalties for non-compliance [266].

#### 3.14.2. Adaptation in the Agriculture Sector

Farmers have a great deal of experience in coping with changes in conditions, including drought and are seen to be one population group with a high capacity to adapt [174,203]. In agricultural systems adaptation to drought occurs through the use of modified or new agricultural practices, such as increasing reliance on crop irrigation and the introduction of drought-resistant crops, as well as diversifying crops and income sources [61,244,254,267,268,269,270,271,272]. With the added challenge of climate change, seasonal climate outlooks are another tool that farmers in the U.S. are turning to in order to better determine which crops will be successful in a particular season [273]. The international literature also highlights the importance of drought forecasts and preparing for drought in advance [243,267]. The Southern African Development Communities Drought Monitoring Centre is an example of such an alerting body [274].

In Canada, agricultural adaptation over the past century has included the development of new agricultural practices and infrastructure to conserve and enhance water supplies and distribution and targeted institutional activities to improve water management [174,203,275]. For example, less frequent tilling and reductions of other field management practices that lowered soil moisture and increased erosion aided in lowering the vulnerability of farmers to the 2001–2002 drought relative to the drought of the 1930s [203,275]. Governments have also increased regional resilience by producing drought plans and enacting legislation to support coordinated water management. In England, water resource management plans have been incorporated into climate change adaptation plans [276]. In the U.S., drought plans have been produced by a number of states [277]. Water management and climate change adaptation plans are being developed by various watershed, municipal and regional authorities across Canada [278,279]. However, climate, human and other factors affecting water sources and reserves are extensive and rarely conform to the jurisdictional boundaries at which watershed management is required [280,281].

Programs to sustain farmers through times of very low production or crop failure due to natural hazards such as drought are expected to be important in the future [282], but developing proactive adaptation actions is key to increasing the resilience of existing agricultural systems to drought [283,284,285,286]. In fact, in the Prairies there are some areas in which conditions such as soil erosion and the accumulation of soluble salts in the root zone may warrant reclassification in use [287]. A media analysis of adaptations undertaken in Canada during the 2001–2002 drought showed that the most frequently mentioned actions included measures related to irrigation, conservation and the management of water resources, insurance and other financial assistance programs (e.g., Canadian Farm Income Program, Net Income Stabilization Account), as well as government based low water responses [288]. Irrigation in particular is one of the most broadly referenced agricultural adaptations to drought in the literature [289]. The use of irrigation systems is increasing across Canada and irrigation is becoming more and more common in regions that had previously relied on rain and subsurface water (Saskatchewan has an Irrigation Strategy to grow and extend the use of irrigation across the province as part of their Water Security Plan http://www.agriculture.gov.sk.ca/Irrigation-Strategy-2014; see also [335]). In Prince Edward Island the growing reliance on irrigation in potato farming and requests for new deep well licenses created serious concerns regarding the sustainability of groundwater supplies and whether or not a moratorium instituted over a decade earlier was still required [290,291]. In November, 2014, a decision was made to uphold the moratorium. In more arid regions where water supplies are finite, like the Okanagan region of southern British Columbia, water demands are managed through a combination of monitoring and management among users (e.g., managing water catchments, use of high elevation dams, reservoirs and instituting seasonal use restrictions). In Alberta’s Oldman Dam water shortages have even been managed using a system of “sharing the shortage” in which users agree to share the reduced allocation of water due to drought [292]. In 2001, partial allotments of 60% were successfully used to ensure the delivery of water supplies to all licensees.

Water consumption can also be reduced by managing the timing of irrigation so that it targets the sensitive stages of crop development. In the case of corn, for example, yield losses may be reduced significantly by preventing water deficits at critical times in development; notably during the reproductive stages [293,294]. Even when plants are subjected to drought conditions, the timing and duration of the event must also be considered since plant processes can often recover following a period of drought if critical management actions are taken at the correct time (e.g., irrigation during flowering). Water conservation strategies and the timing and use of water (water management and irrigation timing) represent simple solutions that can be managed in response to need, when and where required (*i.e.*, the farm scale). In contrast, technological and transgenic approaches will take time to develop [295]. Moreover, since drought resistance is linked to overall plant performance, cultivars that are tolerant to extreme drought conditions may result in reduced production during less extreme or episodic drought conditions [296,297,298]. As such, farmers may need to balance trade-offs between production and the risk of drought in their choice of what to plant next season. Planting seeds representing a diversity of cultivar tolerance may prove beneficial in this case [299]. The combined use of existing drought tolerant cultivars with physiologically sensitive timing of irrigation can serve to reduce water demands during key stages of development. Furthermore, since drought stressed plants and livestock are also more vulnerable to diseases, competition from invasive species and pests, strategies that increase genetic and crop diversity across fields and the landscape may represent adaptations that will provide overall benefits to both health and production [288,300]. Looking to the future, against a global backdrop of diminishing lands suited to agriculture and multiple cropping due to climate change, Canada is expected to experience an increase in the area of land that is marginally or moderately suitable for agriculture in farther northern regions [301].

#### 3.14.3. Adaptation to Build Individual and Community Resilience

At the individual level, personal savings may not be enough to support families during droughts, hence loans, savings strategies and insurance may be important adaptation options [267,270,302]. In the U.S., many families went into bankruptcy under the combined drought and Great Depression in the 1930s and had to draw on government aid for support [202]. Others moved to different communities to earn a living [202]. In 2012, following a particularly dry summer in Ontario, over 5000 crop damage reports were submitted by farmers [303]. Although drought-related crop losses in the Prairies from 2002 were offset by farm insurance [174], not all farmers are registered for insurance [303,304]. Moreover, while smaller farms tend to have more diversified crops which serve in part to mitigate risk (*i.e.*, act as adaptation strategies; [305,306]), they may also be less likely to have insurance coverage.

Another key adaptation to variability has been to increase income from off-farm sources [174] including from oil and gas leases [203]. The long-term viability of such tactics and their impact on social cohesion in communities is unclear. Socioeconomic challenges can impact the adaptive capacity of communities [307], but so can more external influences such as global scale changes in agricultural production, demand and markets [308]. As such, the presence of well-developed social institutions and access to highly qualified personnel and information are particularly important for remote rural communities [309].

In Canada, drought resiliency in some rural and agricultural communities is related to proximity to urban centers. A study by the International Institute for Sustainable Development and Agriculture and Agri-Food Canada [309] in a 550,000 square kilometer prairie region (comprising 53 Census Divisions across Alberta, Saskatchewan and Manitoba) found that the areas exhibiting the highest adaptive capacity index values were clustered within corridors between major urban centers. Proximity to urban centers is thought to bring benefits related to off-farm incomes, access to technology (including computers/computing technologies), information networks (email, Internet) and access to agricultural institutions. Conversely, the lowest adaptive capacity was observed in areas more remote from urban centers, which were situated in more northern zones of this prairie belt. The importance of social networks and connectivity may be more obvious during periods of crisis, such as during the 2002 drought on the Prairies when farmers in eastern Canada who were less affected by drought shipped 64,000 bales of hay to western Canada through “Hay West” to support the worst struck areas [175]. In 2012, some western Canadian farmers attempted to reciprocate with hay to eastern farmers [175]. Adaptations to address the impact of drought on mental health and social capital within the community are also discussed above.

### 3.15. Discussion

#### 3.15.1. Knowledge of Current and Future Drought Impacts on Health

In recent years information about the potential health impacts of drought has increased. Evidence pointing to health impacts that may be modified by climate change has continued to build since earlier reviews (e.g., by Smoyer-Tomic [51] in 2004). The number of Canadian-U.S. studies is increasing, with more than half of the records for this region published after 2004. However, Canadian studies continue to be focused on the Prairies. In addition to studies situated within the Canadian context broadly, more than half of the records identified focused specifically on western Canada and all but one targeted the Prairies specifically. Research linking mental health impacts with wildfire and drought has also expanded significantly. However, research around drought and health remains narrow. Approximately three-quarters of the primary international studies identified were focused on mental health and nearly all focus on Australia. In the Canadian-U.S. literature, more than one-third of the primary studies are focused on mosquito-borne disease, largely WNV and the related SLE. This also appears to be the area of current interest, with these records accounting for more than half of the primary Canadian-U.S. studies published after 2004. Limited data on actual health outcomes results in uncertainty about the severity or magnitude of these impacts. Consequently projections of climate change effects on drought-related health impacts remain difficult to make especially in the Canadian context.

At the same time that more evidence is needed to understand the impact of drought on health outcomes, public health is challenged by the uncertainty surrounding the probable occurrence of future drought in Canada. There has been limited research directed toward the probability and assessment of future droughts specifically over Canada in the context of climate change and variability. Greater certainty around the characteristics of drought anticipated in the future and their linkages with specific health impacts could help adaptation planners better identify and prepare for key risks in their regions, including those related to human health. For example, the literature reviewed in this paper suggests that certain features of drought (e.g., subsequent heavy rainfall events) may significantly increase risks of some infectious diseases among Canadians. Knowledge of geographic areas likely to be affected by drought, as well as potential changes to frequency, duration and severity of events is critical for the development of adaptation strategies which can directly or indirectly reduce health impacts. Coordinated drought research is limited and fragmented in Canada, often carried out only in response to severe drought experience. Although most regions of Canada have experienced drought, the majority of studies have focused on the Prairies due to the greater frequency of droughts in this region. The variety in the forms of drought experienced in Canada needs to be taken into consideration when planning for possible health impacts. Given the complex interactions with water, a discussion of the impact of drought on soil through the health lens may add to the literature on water and water content alone.

#### 3.15.2. Knowledge of Adaptation Options

A recent 2013 systematic review of the effectiveness of public health interventions to reduce the health impacts of climate change highlighted a gap in the literature around adaptation to drought [310]. While there has been some effort to assess agricultural adaptation to drought [311] and public health promotion activities that touch on related impacts, such as spinal cord injury [250,251], none of the public health adaptations specific to drought identified in the international literature had been evaluated. Here, again, a lack of specificity around drought among other climate change-related extreme weather events limits the relevance of these discussions for public health planning.

Potential adaptations to directly or indirectly reduce health risks from drought are plentiful, particularly those that address agricultural impacts of climate change and weather variability. Few adaptations are discussed specifically in the context of drought but are often put forward to address secondary concerns (e.g., vector-borne diseases). Factors influencing agriculture and agricultural practices are complex and sometimes contradictory, ranging from public level issues driven by policy, legislation and international agreements, to environmental and community issues relating to how land is used and the local impacts [287]. To a large extent adaptations to drought are emerging from regional risk and adaptation case studies [282].

There is an opportunity to address multiple climate change-related health impacts under broad public health adaptations. In addition, adaptation activities and programs in other sectors can contribute co-benefits to the agricultural sector. Programs that have been developed for other objectives, such as protecting wildlife or water, have been found to provide incidental benefits to the agricultural sector during drought periods. Such co-benefits are not well recognized, but constitute just short of two-thirds of activities captured in a national inventory of programs for managing drought and agricultural water in Canada [311].

#### 3.15.3. Challenges Adapting to Drought

Measures taken to adapt to drought between 2000 and 2006 over the Canadian Prairies were expensive, their effectiveness varied and challenges were associated with their implementation [199]. Gaps in knowledge and misplaced assumptions have been identified as obstacles to drought adaptation. A number of barriers to agricultural adaptation to drought were highlighted during the drought of 2001–2002 in Canada, including the lack of research and a lack of funds, as well as problems relating to institutions and processes [288]. A study of water management systems in the North American Pacific Northwest found that water management decisions were centred around an assumption of stable conditions and this was compounded by a lack of access to regionally specific climate information [312]. Challenges have also been noted in simply obtaining high quality local hydrologic information from climate change datasets [184,185,313]. The paucity of such information makes it difficult for communities to develop more integrated climate adaptation plans which reduce drought risks.

Severe drought can exceed the adaptive capacity of agricultural producers and communities. In their analysis of the 2001–2002 Canadian drought, Wheaton *et al.* [288] found that some adaptations were ineffective and that drought brought about negative impacts even when adaptation measures were put in place. Drought can be a prolonged event, which also suggests the importance of considering both long-term and short-term adaptations and approaches to mitigate any long-term health impacts.

Despite rural areas having greater social capital than other parts of Canada [176,264], there is much reliance on technologies which may not be able to counter the full impacts of climate change on the production of key crops [178]. Adaptations derived from new technologies will take time to understand, develop and institute (e.g., transgenics or traditional breeding for drought tolerance) [314]. Furthermore, the resources required to generate necessary information about future climate and weather conditions and the required analyses may not be available to provide timely planning direction to farmers, municipalities and other local planners [286,315]. The literature reviewed in this paper highlighted that remote areas and communities may have significantly less adaptive capacity to reduce drought impacts than communities closer to urban centres.

#### 3.15.4. Key Knowledge Gaps

Key knowledge gaps that need to be addressed to inform drought adaptation include:

- Projections of drought for Canada (beyond western Canada) including the features of those droughts and regional susceptibility to increased frequency and/or intensity of major droughts;
- The impacts of drought on infectious diseases which represent a substantial burden of illness in Canada already (e.g., *C. perfringens*, *Campylobacteriosis*), as well as the influence of climate change on these impacts;
- The impacts of drought on disease rates associated with fungi that are now established in Canada (e.g., *Cryptococcus gattii* and *Blastomyces dermatidis*), as well as the influence of climate change on these impacts;
- The impact of drought on particle levels in already stressed airsheds and implications for health, as well as the influence of climate change on these impacts;
- The applicability of mental health impact studies of drought to the Canadian context;
- Impacts of drought on dust and wildfire-related health impacts;
- The effectiveness of adaptations to drought, including the impacts of non-health adaptations (e.g., for the agricultural sector) on reducing negative health outcomes.

## 4. Conclusions

Climate change is now considered to be one of the major threats facing public health in the coming decades [231]. Research on the health impacts of climate change (e.g., extreme heat events, vector-borne diseases) is increasing in Canada and internationally. However, health risks associated with drought and the implications of climate change have been less investigated. To our knowledge, this is the first comprehensive study of the health effects of drought on Canadians and of possible adaptation measures. The direct and indirect health impacts are potentially quite broad and serious given that drought is an important pathway for increased exposure to injuries, air pollution, food, water and vector-borne diseases. Evidence also suggests that a wide range of populations may be at higher risk for these impacts including infants and children, elderly people, the socially and economically disadvantaged, pregnant women, people who spend time outdoors and those with chronic diseases and compromised immune systems.

The possible health impacts of drought on Canadians are of concern because drought is a regular occurrence in some communities. Evidence suggests that the incidences of drought increased over most of the country between 1950 and 2002. Several long duration and severe droughts have affected the southern Canadian Prairies and interior valleys of British Columbia over the last century. Available studies mainly suggest that future droughts will likely be more frequent, longer lasting and more severe in those regions that already experience these events (*i.e.*, more southern and interior areas of the country).

Increased adaptation to drought impacts will be needed at local and regional levels to protect health and to support vibrant and resilient communities. A range of measures exist to address the direct impacts of drought on health or that indirectly modify the ecological or socioeconomic pathways through which health is affected. The nature of individual droughts is expected to influence health impacts. However, there is a paucity of information concerning the effectiveness of many of the health adaptations. Efforts to protect the health of Canadians from future droughts will benefit by increased surveillance and monitoring of health impacts and greater investigation of health risks, vulnerable populations and measures to increase resilience at both the individual and community levels.The results of additional research to address current gaps and support future monitoring and surveillance will be key to inform adaptation and planning for future drought-related health impacts.

## Glossary

Anthrax—Anthrax is caused by an infection from the *Bacillus anthracis* bacterium, which can be found in farm animals [76]. The bacteria can be contracted by humans through the skin, stomach or lungs with the latter associated with the most severe symptoms [76].
Blastomycosis—Blastomycosis is caused by *Blastomyces dermatitidis* fungus [151]. Humans become infected by inhaling the fungal spores, affecting any organ but most commonly the lungs [151]. Infections are rare but may be fatal [151].
*Campylobacter*—Campylobacteriosis is caused by an infection from *Campylobacter* bacteria [76]. Campylobacteriosis affects the gastrointestinal system and may be contracted through the consumption of contaminated food or water, raw milk or close contact with an infected human or animal [76].
Coccidioidomycosis—Coccidiodomycosis, also known as “Valley fever” or “cocci” is caused by *coccidioides* fungus [316]. Humans become infected by breathing in the fungus [316]. Most infected individuals do not experience any symptoms but in some cases, there can be flu-like symptoms and rarely, the infection can be more severe, leading to meningitis or death [316]..
*Clodistrium (C.).perfringens—C. Perfringens* is a bacteria that causes food poisoning through the enterotoxin it produces in the human body[317]. The bacteria is typically found in cooked foods (vegetables, meat, fish, poultry) stored for prolonged periods at room temperatures [317].
*Cryptoccocus gattii—Crytococcus gattii*is a fungus, found on trees and soil that can lead to the illness cryptococcal disease (cryptococcosis) [318]. When the fungus is inhaled, humans can become infected, with impacts on the lungs and may progress to the nervous system [144,318].
Cryptosporidiosis—Cryptosporidiosis is caused by an infection from the *Crytptosporidium* parasites [76]. Cryptosopridiosis is an intestinal infection and is commonly transmitted through water [76].
Eastern equine encephalitis virus (EEEV)—EEEV and Western equine encephalitis virus (WEEV) are part of the genus Alphavirus, with EEE the most severe arboviral encephalitides [319]. EEEV and WEEV are transmitted to humans typically by mosquitoes that feed on animals that have been infected by the virus.
*Escherichia (E.) coli—E. coli* is a group of bacteria found in human and animal intestines that are largely benign [320]. A few strains including *E. coli* O157:H7 can cause (severe) illness in humans (e.g., diarrhea, vomiting, complications such as kidney failure) [320].
Eutrophication—Eutrophication is the excessive growth of aquatic plants (*i.e.*, algae) due to the introduction of nutrients into a body of water such as nitrogen or phosphorous; the availability of these nutrients may previously have been a limiting factor for growth [321]. This excess growth typically alters species composition and reduces the availability of oxygen available to other aquatic life [321].
Giardiasis—Giardiasis is caused by an infection from the parasite *Giardia* and can be contracted from consuming (items contaminated with) fecal matter containing Giardia cysts [76]. Giardiasis affects the intestinal system and is commonly transmitted through water [76].
Hantavirus—Hantavirus can lead to the rare but highly fatal disease Hantavirus pulmonary syndrome [322]. The virus can be transmitted to humans from infected rodents when airborne particles of the infected rodent’s droppings, urine and saliva are inhaled [322]. There have been some accounts of transmission directly from the bites of infected rodents [322].
Hepatitis A—Hepatitis A is caused by an infection from the hepatitis A virus and can be contracted from oral contact with (items contaminated with) fecal matter containing the virus [76]. Hepatitis A is a liver infection and can be prevented by immunization [76].
Leptospirosis—Leptospirosis is caused by the bacteria *Leptospira* [323]. The bacteria can be transmitted from infected animals to humans through contact with water, soil or food that has been contaminated by their urine [323].
Lyme disease—Lyme disease is caused by an infection from the bacteria *Borrelia burgdorferi* [76]. The bacteria *Borrelia burgdorferi* is transmitted to humans by ticks, which feed on both wild animals infected with the bacteria and humans [76].
*Naegleria fowleri—Naegleria fowleri* is an amoeba that destroys the brain in infected individuals [324]. It is present in warm freshwater and soil and infects the human body through the nose [324].
Salmonellosis (*S. Typhi, S. Paratyphi*)—Salmonellosis is caused by an infection from *Salmonella* bacteria [76]. Salmonellosis affects the gastrointestinal system and outbreaks have been associated with a range of foods (e.g., uncooked eggs, meat, poultry, raw produce) [76].
“Silo-filler’s disease”—“Silo-filler’s disease” involves poisoning from NO_2_ gas [158].
St. Louis Encephalitis virus (SLEV)—Humans are typically infected by SLEV by mosquitoes, through the bite of mosquitoes, which have acquired the virus from infected animal hosts (e.g., wild birds) [325].
*Streptobacillus (S.) moniliformis—S. monoliformis* is one of two bacteria that cause “Rat-bite fever” and the form common in North America [326]. Rat-bite fever is transmitted by infected rodents or through the consumption of contaminated food or water [326].
West Nile virus—West Nile virus is part of the family *flaviviridae*, similar to the viruses that cause dengue fever, yellow fever and St. Louis encephalitis [76]. The virus is transmitted to humans by mosquitoes infected with the virus [76].

## Appendix

**Table A1:** Health effects of drought.

Health Effect	Reference (Country/Region)
Food-/Water-borne diseases, water contamination	[Canada, U.S.]
	[23,63,69,75,81,86,110,327] *****; [2,45,46,47,48,49,50,51,52,55,60,62]
	[International]
	[42,58]*****(Africa); [244](Aral sea); [92,109]*****(Australia); [100]*****(Beirut-Lebanon); [227](England and Wales); [65,108] ***** (France); [74] (India); [56] (South Wales) [57]; [99] ***** (Taiwan); [102,103] *****; [44,54,57,59,78,94,208]
Vector-borne and other infectious diseases	[Canada, U.S.]
	[115,116,117,119,121,122,124,125,127,129,131,132,135,140,143,145,146,147,148,328] *****; [46,48,50,51,53,118,142]
	[International]
	[120]*****(France); [141] ***** (Paraguay); [242] (Southern Africa); [130] (UK); [59]
Respiratory impairments	[Canada, U.S.]
	[155,156,158] *****; [46,48,49,80,83,154]
	[International]
	[243](Asia); [162] ***** (Brazil); [161] (Israel); [43]
Malnutrition, Food security	[Canada, U.S.]
	[46,48,50,275]
	[International]
	[42] (Africa); [244] (Aral sea); [243] (Asia); [59,90,206,207,268] (Kenya); [268] (Zimbabwe);[43,44,59,78,90,94,207,208]
Mental health	[Canada, U.S.]
	[51,164,176,258,327]*****; [2,46,50,80,83,164]
	[International]
	[42] (Africa); [196,201,212,213,214,216,217,218,219,221,222,223,224,225,227,228,229,236,254,256,257,259,260,261,263] (Australia) *****; [226,255,329,330] (Australia); [331] ***** (Bolivia); [215] ***** (Brazil); [78,90,207,208,220,332]
Wildfire	[Canada, U.S.]
	[46,48,51,83]
	[International]
	[78]
Injury	[Canada, U.S.]
	[170,171] *****; [23,46,51,153]
Mortality	[Canada, U.S.]
	[23,50]
	[International]
	[243](Asia); [42] (Africa); [268](Zimbabwe); [41,43,44,268]

***** = Primary research.
